# A Digital Intervention for Respiratory Tract Infections (Internet Dr): Process Evaluation to Understand How to Support Self-care for Minor Ailments

**DOI:** 10.2196/24239

**Published:** 2022-01-19

**Authors:** Sascha Miller, Lucy Yardley, Peter Smith, Mark Weal, Alexander Anderson, Beth Stuart, Paul Little, Leanne Morrison

**Affiliations:** 1 Center for Clinical and Community Applications of Health Psychology Department of Psychology University of Southampton Southampton United Kingdom; 2 School of Psychological Science University of Bristol Bristol United Kingdom; 3 Department of Social Statistics and Demography School of Economic, Social and Political Sciences University of Southampton Southampton United Kingdom; 4 Web and Internet Science Group School of Electronics and Computer Science University of Southampton Southampton United Kingdom; 5 Primary Care Research Centre, Primary Care Population Sciences and Medical Unit School of Medicine University of Southampton Southampton United Kingdom

**Keywords:** illness behavior, self-care, internet, evaluation studies, respiratory tract infection, mobile phone

## Abstract

**Background:**

Approximately 57 million physician appointments annually in the United Kingdom are for minor ailments. These illnesses could be self-cared for, which would potentially lower patients’ anxiety, increase their confidence, and be more convenient. In a randomized controlled trial of the *Internet Dr* digital intervention, patients with access to the intervention had fewer consultations for respiratory tract infections (RTIs). Having established intervention efficacy, further examination of trial data is required to understand how the intervention works.

**Objective:**

This paper reports a process evaluation of *Internet Dr* usage by the intervention group. The evaluation aims to demonstrate how meaningful usage metrics (ie, interactions that are specific and relevant to the intervention) can be derived from the theoretical principles underlying the intervention, then applied to examine whether these interactions are effective in supporting self-care for RTIs, for whom, and at what time.

**Methods:**

The *Internet Dr* trial recorded patients’ characteristics and usage data over 24 weeks. At follow-up, users reported whether their levels of enablement to cope with their illness changed over the trial period. The Medical Research Council process evaluation guidance and checklists from the framework for Analyzing and Measuring Usage and Engagement Data were applied to structure research questions examining associations between usage and enablement.

**Results:**

Viewing pages containing advice on caring for RTIs were identified as a meaningful metric for measuring intervention usage. Almost half of the users (616/1491, 42.31%) viewed at least one advice page, with most people (478/616, 77.6%) accessing them when they initially enrolled in the study. Users who viewed an advice page reported increased enablement to cope with their illness as a result of having participated in the study compared with users who did not (mean 2.12, SD 2.92 vs mean 1.65, SD 3.10; mean difference 0.469, 95% CI 0.082-0.856). The target population was users who had visited their general practitioners for an RTI in the year before the trial, and analyses revealed that this group was more likely to access advice pages (odds ratio 1.35, 95% CI 1.159-1.571; *P*<.001).

**Conclusions:**

The process evaluation identifies viewing advice pages as associated with increased enablement to self-care, even when accessed in the absence of a RTI, meaning that dissemination activities need not be restricted to targeting users who are ill. The intervention was effective at reaching the target population of users who had previously consulted their general practitioners. However, attrition before reaching advice pages was high, highlighting the necessity of prioritizing access during the design phase. These findings provide guidance on how the intervention may be improved and disseminated and have wider implications for minor ailment interventions.

## Introduction

### Background

Minor ailments are defined as nonserious health conditions that may be cared for by patients (eg, back pain, respiratory tract infections [RTIs], headache, and stomach upsets) [1]. However, an estimated 57 million unnecessary visits to general practitioners (GPs) in the National Health Service (NHS) occur every year in the United Kingdom as patients seek advice for managing these conditions [2]. The strain this places on primary care resources is well documented [3,4]; however, there is also a cost to the patient through increased anxiety, lowered confidence, and inconvenience [1]. Promoting self-care for these ailments would help alleviate the stress on both primary care and patients by helping patients understand and feel more enabled to cope with their health [3].

Many patients already use web-based resources for guidance with health issues [5]. However, credible, evidence-based interventions are needed to ensure that potentially serious infections are identified, and users are advised to consult a health care professional (HCP) when necessary. Interventions aimed solely at increasing users’ knowledge regarding their illness have shown only limited effects on increasing self-care [4]. Instead, calls have been made for interventions that address barriers to self-care, such as patients feeling distressed regarding their symptoms and not knowing how to treat them [4,6]. Theoretically-based digital health interventions have the potential to address these barriers and offer the advantage of providing ongoing support at a time and place that is convenient to the user. *Internet Dr* is a digital intervention that supports appropriate self-management of RTIs [7]. The intervention content is theoretically underpinned and contains tailored advice on self-caring for RTI symptoms, as well as a symptom checker to identify serious illnesses, including meningitis and sepsis [6] (see the *Intervention* subsection in the *Methods* section for more details). The content was designed to address previously identified barriers to self-care: (1) uncertainty regarding the need for medical treatment and (2) distress caused by the symptoms [4,6].

A randomized controlled trial (RCT) of *Internet Dr* was conducted over the winters of 2012 and 2013, with 3044 participants recruited randomly from lists of all patients registered at a selection of general practices in southern England. Users who had access to the intervention had fewer GP consultations for an RTI compared with those in the control group (239/1574, 15.18% vs 304/1664, 18.26%; multivariate risk ratio 0.71, 95% CI 0.52-0.98; *P*=.04), despite both groups having an equivalent occurrence of illnesses [7]. This means that more users in the intervention group decided to self-care for their symptoms. In addition to GP visits, self-reported scores for the patient enablement index (PEI) were also collected as an outcome measure at follow-up to capture the psychological benefits for patients using the intervention [8]. The PEI items asked users to reflect on perceived changes that occurred as a result of having participated in the study; for example, “thinking about the kinds of symptoms we have asked about in this study, compared with before you took part in this study, do you feel you are able to help yourself: same or less; better; much better?” Having previously focused on the intervention’s impact on health service use [7], a process evaluation of the RCT data is required to understand the psychological changes and behavioral engagement with the theoretically underpinned content of the intervention that led to the intervention group’s increased ability to self-care.

### Objectives

Process evaluations aim to provide insight into the parts of an intervention that work, for whom, and under what conditions [9,10]. This may be achieved by examining the underlying intervention mechanisms that are anticipated to lead to positive outcomes and the impact of context on the implementation of an intervention [9,10]. Logic models are often used to map the intervention content, theoretical underpinning, anticipated mechanisms of action, and outcomes [11], thereby identifying core research questions or hypotheses to address within a process analysis [12]. By explaining the mechanisms and effects of context, process evaluations have the potential to inform future intervention development and dissemination and advance our understanding of intervention theory [9,10].

Quantitative usage data collected automatically during interactions with a digital intervention (ie, log data) have the ability to provide a rich source of metrics for usage analyses [13-16]. Although widely used, broad, summative measures of usage, such as time spent or number of pages or components viewed in an intervention, have been criticized for their inability to explain how usage leads to positive outcomes [15,17-20]. In addition, the breadth of potential usage metrics available means that there is a danger that inferential analyses that examine all of these variables will produce results that do not relate meaningfully to the theoretically designed intervention architecture and are therefore unable to offer specific practical and actionable recommendations to optimize future intervention designs [18]. A clear rationale for choosing usage metrics is necessary to understand what is being measured and what can be inferred from analyses [19,21,22]. On that basis, arguments have been made to identify usage metrics that are meaningful to the intervention rather than data dredging [15]. This means determining types or patterns of usage that are specific to an intervention’s structure and the target behavior and are able to examine usage of theory-based content. For example, by isolating usage of a specific component [15,18]. For example, by isolating the use of a specific component or set of pages aimed at improving users’ self-efficacy for carrying out a target behavior, it is possible to examine the relationships between having viewed that component, reported changes in self-efficacy, and behavioral outcomes.

The framework for Analyzing and Measuring Usage and Engagement Data (AMUsED) [15] was developed to support systematic usage analyses of digital interventions by guiding researchers through 3 stages of planning and carrying out analyses. Stage 1 focuses on familiarization with the intervention architecture, including content, structure, and data collection. Through a list of generic questions in the first section of stage 2, researchers identify available metrics with which to measure usage, covering both summative measures (eg, number of times the intervention was accessed, completing the intervention, and amount of time spent) and more in-depth measures (eg, type, frequency, and completion of theoretically-based content). Researchers are then encouraged to consider these variables alongside the information from stage 1 and identify usage metrics that are relevant to the intervention structure, theory-based content, and target behavior and that are most likely to provide insight into how the intervention was effective and may be improved and implemented. Sections 2 and 3 of stage 2 then address how these metrics may be used in inferential analyses with self-report measures for user characteristics and target behaviors or outcomes. Stage 3 focuses on planning compatible data collection to ensure that analysis using appropriate analytical software is both possible and less onerous. The framework has previously been used to shape data collection for other digital interventions [15]; however, this process evaluation reports the first application to a usage analysis.

In line with the Medical Research Council guidance, this paper reports the process evaluation of the *Internet Dr* RCT, including a detailed usage analysis structured by the AMUsED framework [15]. The aims of the evaluation are (1) to identify measures of usage that are meaningful to the intervention and (2) to examine which parts of the intervention worked, for whom, and at what time. These results will generate guidance on how the design, implementation, and dissemination of *Internet Dr* can be improved but will also have generic implications for guiding the successful design and development of other digital interventions promoting self-care for minor ailments.

## Methods

### Internet Dr Trial Design

An open, pragmatic, parallel-group RCT of the *Internet Dr* digital intervention had been previously conducted [7]. After completing web-based enrollment in the study and baseline measures, participants were randomized using computer-generated random numbers to either the intervention group who had access to the website or the control group who did not. Having completed outcome measures at 24 weeks, participants in the control group were able to view the intervention. The full details of the *Internet Dr* RCT and findings from the primary analysis are available in the study by Little et al [7]. The study was registered with a trial registration number of ISRCTN91518452, and ethics approval from the South West Medical Research Ethics Committee, United Kingdom Health Departments’ Research Ethics Service, was obtained.

### Participants

Adults (aged ≥18 years) registered with GPs within NHS Primary Care were recruited for the RCT by postal invitation. Patients with severe mental health problems or terminal illnesses were excluded. Participants needed to have access to the internet, with only 1 participant per household taking part. The process evaluation only examines participants who were randomly allocated to the intervention group and therefore had access to the intervention during the 24-week trial period.

### Process Evaluation Design

A plan for conducting a complete process evaluation of data collected during the *Internet Dr* RCT was designed and conducted in line with the Medical Research Council guidance [12] using the AMUsED framework [15]. On the basis of the AMUsED framework checklists, the intervention’s structure, theoretical underpinning, and data collection points were collated (stage 1; Multimedia Appendix 1 [7,8,22-29]). All available usage metrics were considered in relation to the information in stage 1 to ascertain the types of usage that would be most meaningful to the intervention (stage 2, section 1; Multimedia Appendix 2). A comprehensive list of research questions was then generated to examine associations between the meaningful measures of usage, user characteristics, and outcomes (stage 2, sections 2 and 3; Multimedia Appendix 2). The questions were refined based on the logic model (see the *Intervention* section). The most appropriate analytical tools for examining the research questions were selected, and the necessary data preparation was considered (stage 3; Multimedia Appendix 3).

The process evaluation team combined expertise in psychology, primary care, statistical analyses, and computing. A total of 4 team members were previously unfamiliar with the intervention. The other 4 researchers had been involved in various stages of the *Internet Dr* development and primary outcome evaluation of the trial [7] and advised on the intervention content, logic model, and data capture processes and analyses. The first author (SM) had previously developed the AMUsED framework for application in process evaluations but was not familiar with the *Internet Dr* intervention.

### Measures

Participants were requested to complete web-based baseline measures at the start of the trial, interim questionnaires every 4 weeks on RTI occurrence, and outcome measures at 24 weeks. Actual GP visits before and during the trial were collected after 1 year from participants’ GP records. Log data for individual users and sessions were collected during the trial (eg, pages accessed, time spent, and order of pages viewed).

Modifiable psychological characteristics thought to underlie decisions to self-care were measured at baseline and follow-up to capture any changes over the trial period that may help to explain outcomes (theory of planned behavior [TPB]) [22]: attitudes and norms; perceived behavioral control (PBC); and beliefs regarding the necessity of HCPs: health locus of control [23] and Krantz Health Opinion Survey [24]). Trait anxiety (Health Anxiety Inventory [25]) and intentions to use and follow intervention advice (TPB) [22] were measured at baseline. Experiences of accessing and using the intervention were collected at follow-up (Problematic Experiences of Therapy Scale [PETS]; [26]), along with the psychological outcome measure of how much users felt their ability to cope with an RTI had changed over the course of the trial (PEI; [8]). Full details of the psychological measures and response items are available in Multimedia Appendix 4 [8,16,19, 22-24].

### Internet Dr Intervention

#### Overview

*Internet Dr* is a web-based digital intervention developed using LifeGuide software (University of Southampton) [30]. All participants were encouraged to log in as soon as they received the invitation letter to the study from their GPs. Having completed trial enrollment and baseline measures, participants allocated to the intervention group were able to access the entire intervention immediately and at any point throughout the study. In addition to completing the interim questionnaires, users were encouraged to log in again if they experienced an RTI. The intervention was developed between 2008 and 2009 before the widespread use of smartphones.

#### Intervention Content

**Figure 1:**
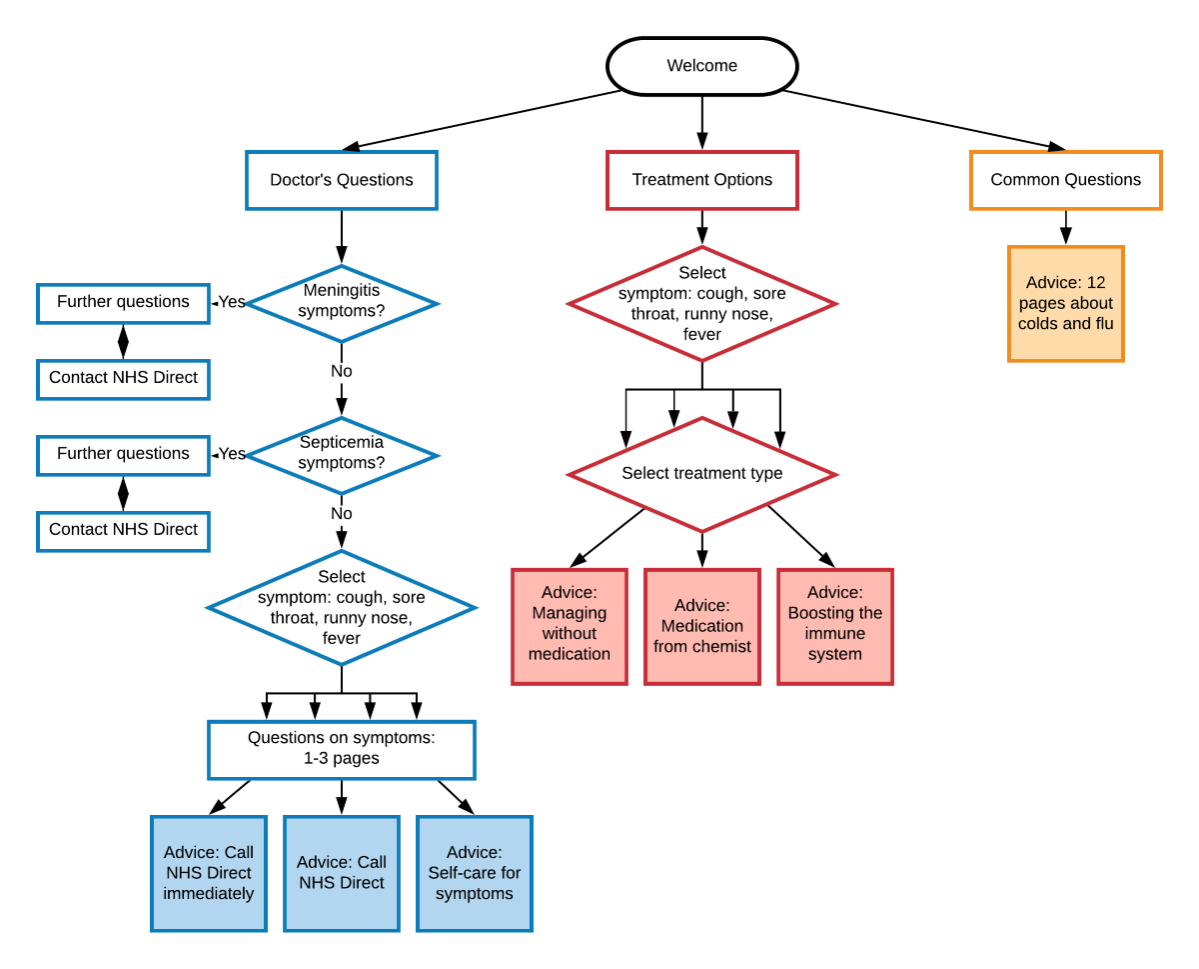
Page flow of the 3 components in Internet Dr leading to advice pages. Oval: start; rectangle: input pages; diamond: decision pages; shaded: advice pages; arrows: direction of movement. NHS: National Health Service.

**Figure 2:**
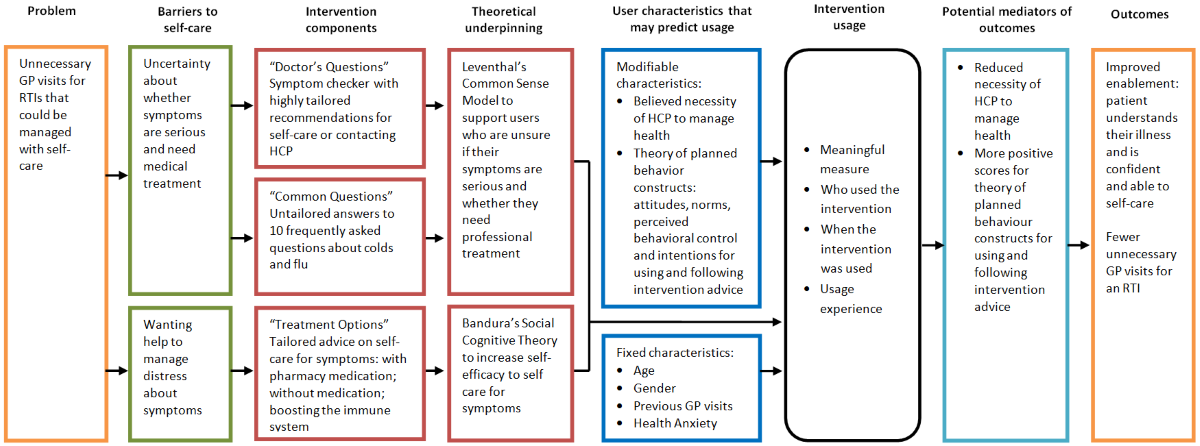
Logic model for Internet Dr intervention. GP: general practitioner; HCP: health care professional; RTI: respiratory tract infection.

*Internet Dr* comprises 3 theory-based components offering varying levels of tailored advice (Figures 1 and 2). Full details and examples of content are available in the study by Yardley et al [6]. The *Doctor’s Questions* component contains a symptom checker with detailed questions regarding users’ symptoms. On the basis of these answers, users are shown 1 of 3 tailored advice messages: (1) “Your symptoms could be a sign of a serious condition that needs urgent care, ring NHS Direct immediately”; (2) “You should contact NHS Direct for further advice”; and (3) details on how to self-manage symptoms with a recommendation to revisit the website should their symptoms not improve or deteriorate further. NHS Direct was a triage phone service where patients were advised whether they needed to visit a hospital or their GP for their symptoms, which has since been replaced by NHS 111. Where patients are recommended by NHS Direct to contact their GP, this information is not automatically transferred to the patient’s GP notes. This component of the intervention ensures that users with potentially serious infections receive the required treatment. The *Common Questions* component provides answers to 10 frequently asked questions regarding RTIs (eg, “how can I tell if my symptoms are due to a cold or flu?”). Questions of interest are chosen by the user; however, there is no tailoring in the answers provided. Both components are informed by Leventhal’s common sense model of self-regulation of health and illness [27] and aim to support users who are unsure whether their symptoms are serious and they need medical treatment.

The *Treatment Options* component supports users in managing any distress they have regarding their symptoms. The content is informed by Bandura’s social cognitive theory [28] to increase users’ self-efficacy in managing their symptoms independently. This section offers tailored advice on self-managing an RTI dependent on the symptom selected and preferred type of treatment (ie, without medication, medication from pharmacy, and boosting the immune system). Although this component offers advice based on the type of treatment selected by the user, it is less tailored than *Doctor’s Questions*, where the advice is specific to the individual and their need to consult the NHS.

Although each component is structured around a psychological theory and aimed at a specific barrier to self-care, the advice pages draw upon common behavior change techniques [31]. These include instructions on how to perform the behavior, information regarding health consequences, and regulation through pharmacological support and by reducing negative emotions.

#### Intervention Structure

All 3 intervention components are designed such that users are required to navigate through specific pages before reaching the RTI management advice (Figure 1). However, each component is structured differently with varying numbers of prerequisite pages, therefore, requiring differing levels of effort to access advice. For example, because of the high level of tailoring required for the symptom checker, a user may view up to 10 pages of questions within *Doctor’s Questions* before reaching an advice page. In contrast, users accessing *Common Questions* need only view 1 page before accessing advice. The advice pages are designed to be standalone, such that viewing a single page from any component may provide the user with the necessary support for self-care.

#### Intervention Logic Model

The logic model illustrates the barriers to self-caring for RTI symptoms that are suggested to influence unnecessary GP visits (Figure 2). Use of the theoretically underpinned content was anticipated to change modifiable characteristics underlying those barriers to self-care, leading to increased levels of enablement. For example, the constructs of the TPB (Figure 2) [22] were measured to capture attitudinal and normative beliefs regarding using and following the intervention advice.

On the basis of the logic models, the following are hypothesized:

- Hypothesis 1: Meaningful usage of the intervention is associated with higher levels of enablement.
- Hypothesis 2: Baseline user characteristics predict meaningful intervention usage.
- Hypothesis 3: Changes in modifiable user characteristics mediate the relationship between meaningful usage and enablement.

### Statistical Analysis

All analyses examined data from only the intervention group. The LifeGuide Visualization Tool (University of Southampton) [32] was used to examine which pages had been accessed, at what point in the trial, and the number of users who had viewed them. Statistical analyses were conducted using SPSS for Windows (version 24; IBM Corp). All validated scales were used and scored according to the published guidance. Higher scores are indicative of positive change in all measures. Changes in scores for modifiable characteristics were calculated by subtracting individuals’ scores at baseline from their scores at follow-up. The frequency distribution of scores for constructs was visually assessed for normality; where these were inconclusive, Kolmogorov-Smirnov tests were performed. All scales were nonnormally distributed except for change scores. As each advice page was designed to be effective in isolation, as opposed to having an additive effect, a dichotomous categorical variable for users who viewed or did not view advice pages was calculated. All analyses were 2-tailed. Owing to a data collection error, it was not possible to analyze responses for the TPB construct of attitudes to using and following the advice at baseline and follow-up.

The distribution of scores and residuals for the PEI was positively skewed. Therefore, regression analyses were not possible for the PEI. Subsequently, 95% CIs were conducted to examine mean differences (MDs) in enablement based on usage and point biserial Spearman ρ correlation coefficient to examine the relationships between changes in user characteristics and enablement. Differences in scores for PETS based on usage were also examined using 95% CIs. Logistic regression was used to examine whether user characteristics at baseline predicted usage. Simple linear regression analyses were performed to examine whether usage predicted changes in modifiable characteristics.

## Results

### Intervention Group Characteristics

A total of 31 general practices invited 43,769 patients to take part in the RCT. Of these 43,769 patients, 3044 (6.95%) consented to take part. Of the 3044 patients, 121 (3.98%) participants left their practice over the course of the study, leaving a total of 2923 (96.02%) users; of the 2923 participants, 1491 (51.01%) were in the intervention group, and 1432 (48.99%) were in the control group (Table 1). The patients’ GP notes showed that 18.04% (269/1491) of people in the intervention group had visited their GP for an RTI in the year before the study. Over the course of the trial, 57.14% (852/1491) of participants in the intervention group reported having an RTI.

**Table 1:** User characteristics and psychological measures collected on the web and usage data (N=1491).

Measure				Baseline					Follow-up		
				Values, n (%)		Values, mean (SD; range)		Values, n (%)			Values, mean (SD; range)
**User characteristics**											
	Age (years)			1490 (100)		56.78 (13.52; 18-89)		—^a^			—
	Female			816 (54.77)		—		—			—
**Psychological measures**											
	Health anxiety			1491 (100)		8.20 (4.65; 0-34)		—			—
	**TPB^b^**										
		Subjective norm	1387 (93.02)		9.07 (2.85; 0-14)		833 (55.87)			8.50 (2.72; 0-14)	
		PBC^c^	1426 (95.64)		10.20 (2.85; 0-14)		831 (55.73)			8.07 (3.63; 0-14)	
		Intentions	1445 (96.91)		9.37 (3.40;0-14)		—			—	
	Health locus of control			1487 (99.73)		13.90 (5.12; 0-21)		962 (64.52)			13.54 (5.05; 0-21)
	Krantz Health Opinion Survey			1490 (99.93)		27.45 (9.15; 0-49)		966 (64.79)			27.35 (8.98; 0-49)
	**PETS^d^**			—		—					
		Made symptoms worse					458 (30.72)			4.34 (0.88; 1-5)	
		Uncertain how to use intervention					458 (30.72)			4.37 (0.95; 1-5)	
		Doubts about intervention efficacy					458 (30.72)			4.03 (1.08; 1-5)	
		Practical problems					458 (30.72)			4.19 (0.96; 1-5)	
	PEI^e^			—		—		952 (63.85)			1.86 (3.03; 0-12)
**Summative usage data**				—		—					
	Number of log-ins							1491 (100)			4.86 (2.87; 0-18)
	Time spent (minutes)							1491 (100)			4.68 (6.57; 0-44.58)
	Number of pages viewed							1491 (100)			10.10 (10.99; 0-81)
**Meaningful usage data**				—		—					—
	Viewed any advice							616 (41.32)			
	Viewed Doctor’s Questions advice							244 (16.37)			
	Viewed Treatment Options advice							297 (19.92)			
	Viewed Common Questions advice							372 (24.95)			

^a^Not collected.

^b^TPB: theory of planned behavior.

^c^PBC: perceived behavioral control.

^d^PETS: Problematic Experiences of Therapy Scale.

^e^PEI: patient enablement index.

### Describing and Defining Usage

#### What Type of Usage Is Meaningful to the Intervention?

Summative measures of usage for the number of log-ins, time spent on the intervention, and number of pages viewed were examined (Table 1). The number of log-ins includes completing interim questionnaires every 4 weeks (when intervention content may not have been viewed). The number of pages viewed and time spent on the intervention varied depending on the size and required interaction for each component (Figure 1). In addition, the only intervention pages that contained theory-based behavioral change techniques were the advice pages in each of the 3 components, meaning that only users who viewed an advice page received support in self-caring for their symptoms. On this basis, having viewed an advice page from any of the components was considered a meaningful way of examining usage.

#### How Many People Reached Advice Pages and When Were They Viewed?

**Figure 3:**
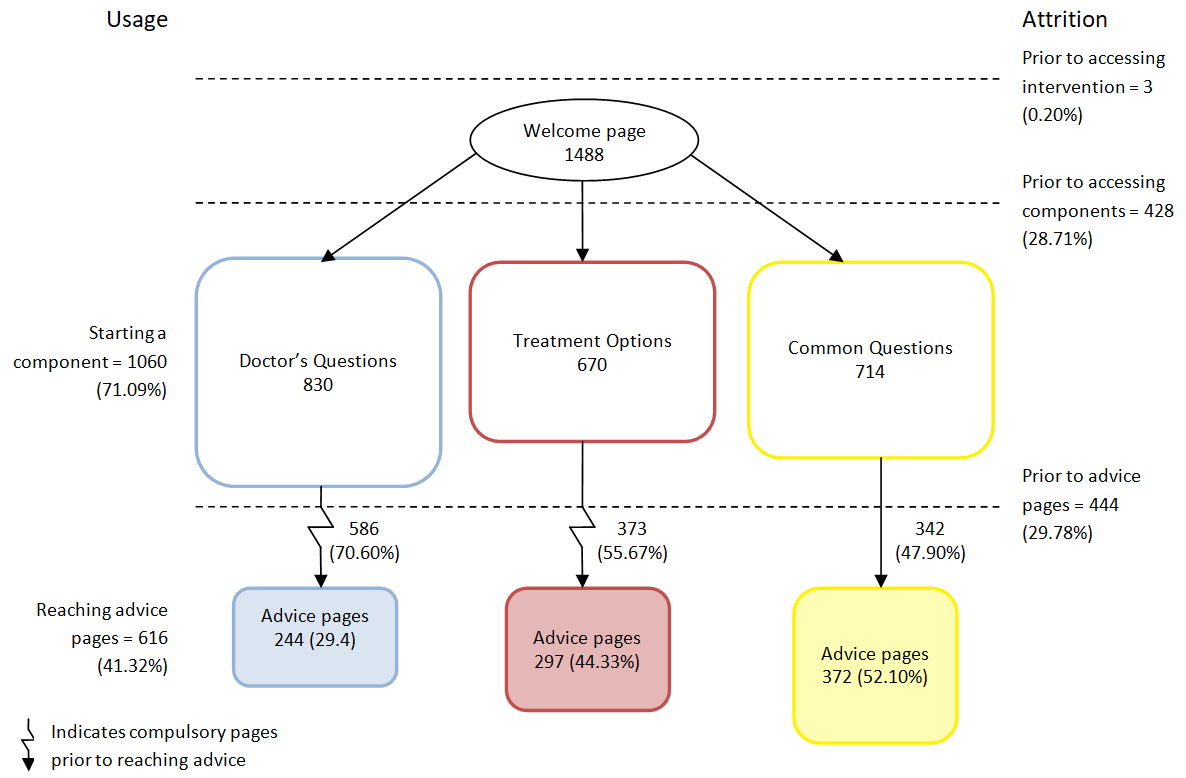
Numbers of users reaching or leaving components and advice pages.

Approximately 42.32% (616/1491) of users viewed at least one of the advice pages (Figure 3; Table 1). For views by component, a total of 913 views indicated that almost half of the 616 users viewed >1 component (297/616, 48.2%). The level of attrition before accessing the 3 components (428/1491, 28.71%) was similar to the attrition within each component before reaching an advice page (444/1491, 29.78%). *Doctor’s Questions*, the component with the most pages, was accessed by most users and saw the highest attrition. Of the users who viewed advice pages, the highest proportion (478/616, 77.6%) did so during their first log-in, having just completed the baseline questionnaire.

#### Did Usage Experiences Differ for Users Who Viewed or Did Not View Advice Pages?

Scores for PETS [23] at 24 weeks revealed that users who had not viewed advice pages were more likely to report that the intervention made their symptoms worse (MD 0.260, 95% CI 0.100-0.420); they were uncertain regarding how to use the intervention (MD 0.289, 95% CI 0.116-0.462); and they experienced more practical problems that prevented them from accessing the intervention, such as forgetting or being too tired or busy (MD 2.57, 95% CI 0.082-0.431). Users who had viewed advice pages and those who had not held equivalent positive beliefs regarding the efficacy of the intervention (MD 0.161, 95% CI −0.038 to 0.361).

### Testing the Logic Model

#### Hypothesis 1: Viewing Advice Pages Predicts Increased Enablement

Users who viewed an advice page were more likely to report increased understanding and ability to cope with their illness as a result of having participated in the study compared with users who did not view advice pages (Table 2). When examined by individual components, the difference in enablement scores between users who viewed an advice page from *Treatment Options* and those who did not was great enough to be practically significant.

**Table 2:** CIs comparing scores for enablement at follow-up between users who did or did not view advice pages (n=952).

Component viewed	Not viewed advice pages			Viewed advice pages			Mean difference (95% CI)
	Values, n (%)	Values, mean (SD)	Values, n (%)		Values, mean (SD)		
Any	532 (55.9)	1.65 (3.10)	420 (44.1)		2.12 (2.92)	0.469 (0.082 to 0.856)	
Doctor’s Questions	785 (82.5)	1.78 (3.04)	167 (17.5)		2.22 (2.97)	0.432 (−0.074 to 0.938)	
Treatment Options	755 (79.3)	1.68 (2.99)	197 (20.7)		2.55 (3.08)	0.875 (0.402 to 1.348)	
Common Questions	683 (71.8)	1.79 (3.12)	269 (28.3)		2.04 (2.79)	0.258 (−0.170 to 0.686)	

#### Hypothesis 2: Baseline User Characteristics Predict Viewing Advice Pages

Users were more likely to view advice pages if they had stronger intentions to use and follow the website advice and stronger beliefs in their ability to self-manage their illness (Table 3). The number of times a user had visited their GP for an RTI in the year before the trial was the strongest predictor for viewing advice pages. Other characteristics measured at baseline were not predictive of viewing advice pages.

**Table 3:** Variables predicting viewing or not viewing advice pages.

Predictors		β (SE)	Wald test (*df*)	*P* value	Odds ratio (95% CI)
Age		−.003 (0.004)	0.5 (1)	.47	0.997 (0.988-1.005)
Gender		−.037 (0.118)	0.1 (1)	.75	0.963 (0.764-1.215)
Health anxiety		.020 (0.012)	2.7 (1)	.10	1.020 (0.996-1.045)
**Theory of planned behavior**					
	Subjective norms	−.008 (0.023)	0.1 (1)	.72	0.992 (0.948-1.037)
	PBC^a^	.021 (0.028)	0.6 (1)	.44	1.022 (0.967-1.079)
	Intentions	.064 (0.023)	7.5 (1)	.006	1.066 (1.018-1.116)
Health locus of control		.011 (0.013)	0.8 (1)	.37	1.011 (0.987-1.036)
Krantz Health Opinion Survey		.018 (0.007)	6.7 (1)	.009	1.018 (1.004-1.032)
Past general practitioner visits		.300 (0.078)	14.9 (1)	<.001	1.349 (1.159-1.571)

^a^PBC: perceived behavioral control.

#### Hypothesis 3: Changes in Modifiable User Characteristics Mediate the Relationship Between Viewing Advice Pages and Enablement

Viewing advice pages did not predict changes in any of the measured modifiable user characteristics over the trial period (norms: *F*_1,784_=0.117; *P*=.73; PBC: *F*_1,798_=1.089; *P*=.30; health locus of control: *F*_1,957_=0.142; *P*=.71; Krantz Health Opinion Survey: *F*_1,964_=1.037; *P*=.31). Changes in these modifiable characteristics do not mediate the observed association between viewing advice pages and enablement.

Strengthened normative beliefs (ie, that friends and family support using and following intervention advice) and increased perceived ease of using and following the advice over the trial period were positively correlated with enablement (*r****_s_***=0.140; *P*<.001 and *r****_s_***=0.269; *P*<.001, respectively). No relationships were observed between enablement and changes in reported dependence on HCPs (health locus of control: *r****_s_***=0.024; *P*=.47; Krantz Health Opinion Survey: *r****_s_***=0.003; *P*=.91).

## Discussion

### Principal Findings

This paper presents a process evaluation of data previously collected in an RCT of the *Internet Dr* intervention designed to enable users to appropriately self-care for RTIs [7]. The aims of the evaluation were to identify meaningful measures of usage (ie, types or patterns of interaction that are relevant to the structure and theory-based content of the intervention) with which to undertake a systematic process analysis and to examine the parts of the intervention that worked, for whom, and in what context.

This evaluation provides a clear example of when summative measures of usage (eg, number of log-ins and time spent on the intervention) would not provide the fine-grained details necessary to understand how the intervention worked; instead, it identifies usage metrics that are relevant to the structure and theory-based content of *Internet Dr*. With regard to the number of log-ins, these include users who logged in to complete interim study questionnaires as well as users who were accessing the intervention. Therefore, inferential analyses using the number of log-ins would capture users’ engagement with the trial as well as with the intervention. Alternatively, if the number of pages viewed had been analyzed and found to be associated with increased enablement, it is unclear what this would have meant or how it may be applied to improve the intervention. Each of the components varied in size, so that users who chose the *Doctor’s Questions* component may have viewed 5 pages and then left the component without having reached any advice on self-caring (Figure 1). In comparison, if users who visited the *Common Questions* component also viewed 5 pages, they would have been able to access 4 pages of advice. Without knowing what the content of the pages is, the number of pages viewed provides little insight into how users experienced increases in enablement or for improvements to the website. Instead, as the advice pages are the only content aimed at supporting users to self-care for their illness, viewing advice pages from the different components was identified as the most meaningful metric with which to analyze the use of the intervention. Users who viewed any advice page were more likely to report higher levels of enablement at 24 weeks compared with users who did not. Although this effect was fairly small, when analyzed by individual components, it was apparent that viewing an advice page from *Treatment Options* led to a practically significant increase in enablement. Therefore, viewing an advice page represents the minimal type and amount of usage required to improve outcomes and may be described as *effective engagement* with the intervention [18].

The 3 components were initially accessed by similar volumes of users, with almost half accessing >1 component. This suggests that offering a variety of content may be useful to maximize the number of users who reach the key features of an intervention. Most users (478/616, 77.6%) who viewed advice pages did so during their first log-in, having just completed the baseline questionnaire. Given that this figure represents more than half of all users in the intervention group who reported having an RTI during the 24-week trial (852/1491, 57.14%), it is unlikely that everyone accessing advice was experiencing an RTI at that precise time. Most if not all users will have experienced RTIs in the past and become familiar with any symptoms they found challenging. This prior experience could have supported *well* users to engage fully with the intervention and access pertinent advice without having to experience the symptoms at that point. This would also be the case for many other common ailments, which suggests that promoting intervention usage when users are well would be effective for self-caring for future minor ailments, as seen with RTIs.

As past behavior is typically a strong predictor of future behavior [33], and previous GP visits reinforce a patient’s decision to return to the GP in future [1], it was anticipated that users who had consulted their GP for an RTI in the year before the study would be less likely to use the intervention and view advice pages. However, the process analysis shows the reverse to be true, with these target users being more likely to view advice pages. As using *Internet Dr* has already been shown to lower the number of GP visits [7], it is probable that reaching these users was key to achieving this.

Viewing advice pages is important for increasing users’ enablement to self-care, with *Treatment Options* advice showing the greatest impact. However, users’ characteristics measured across the study provide only a limited explanation of the psychological changes that led to better enablement. *Internet Dr* content is underpinned by social cognitive theory (*Treatment Options*) and Leventhal’s common sense model [27] (*Doctor’s Questions* and *Common Questions*). The TPB was selected, along with measures of beliefs regarding the necessity of HCPs to manage illness to measure psychological changes across the study. Azjen cites Bandura’s definition of self-efficacy as the basis for the construct of PBC within the TPB [34]. Therefore, users who accessed *Treatment Options* content based on social cognitive theory were expected to report increased PBC; however, this effect was not found. Bandura [35] stipulates the use of measures of self-efficacy with social cognitive theory, and considering that PBC encapsulates several constructs [36], a general self-efficacy measure may be preferable for future studies of this nature [37]. Since the development of *Internet Dr* in 2008, further research has identified additional barriers to self-care (eg, perceptions of illness severity, not considering alternative options, and cost implications of paying for unprescribed medication [4]). Although these were not intentionally targeted within *Internet Dr*, the increases in enablement and the lower GP visits suggest that the theoretically underpinned content may have been effective in addressing some of these additional barriers. Future research, including measures of user characteristics that better reflect these barriers, may provide a more in-depth explanation of the association between usage and enablement.

In addition to examining users who viewed advice pages, it is also important to consider that approximately two-thirds of users in the intervention group did not access any advice pages. The first point of attrition for these users occurred at the *Welcome Page,* with just under one-third of users not progressing any further (Figure 3). An explanation for this might be that many of these users were not ill at the time and intended to return to the intervention if they experienced an RTI. However, these findings suggest that using advice pages when users are well can still be of benefit. Users who did not view advice pages were also more likely to report practical barriers to usage (eg, too tired or busy and forgetting). Applying these findings to future dissemination means that patients can be encouraged to access the intervention at a time that is convenient to them and not have to wait until they are experiencing symptoms. This could be reiterated to users by adding a message to the *Welcome Page*.

The second incidence of attrition occurred within the components, with almost another one-third of users starting a component but not reaching an advice page. The highest proportion of this attrition occurred in the *Doctor’s Questions* component, which is the largest component. This section includes a compulsory symptom checker with up to 10 pages of questions necessary in any intervention for minor ailments to ensure that serious infections in need of urgent medical attention are identified. Although these questions could not be omitted, the format and layout could be amended to minimize the burden on the user and subsequent attrition. For example, streamlining content by combining pages or motivating users to continue by including page numbers or breadcrumbs to show progress and location may have lowered attrition. Interestingly, these compulsory pages were not raised as a concern in qualitative evaluations conducted during the development phase [6]. This highlights the ability of process evaluations to establish design precedents from post hoc data analysis, such as ensuring that users are motivated and able to access the active ingredients of interventions with minimal effort.

### Limitations

Scores for increased enablement were low, with most users selecting *same or less* (0), which probably reflects that most users have experienced and successfully self-cared for RTIs previously. This is supported by the finding that users who had failed to self-care before the study were more likely to use advice pages. The PEI scoring was problematic as there were only 3 response options available, which did not allow users to distinguish between no change and deterioration in enablement. The resultant skew in scores meant that regression analyses were inappropriate for examining variables predicting enablement. Recent studies have measured PEI using Likert scales of ≥5, allowing for multiple, finer-graded levels of response [38,39].

The purpose of this study was to examine the psychological outcomes of intervention users and to explain the effectiveness of the intervention. As a result of the problematic PEI scoring, we considered examining the relationship between the usage of advice pages and the behavioral outcome of GP visits. However, this was not possible for several reasons. Almost half of the users who had viewed an advice page accessed advice from >1 component, and some users viewed several advice pages within a component, meaning that it was not possible to match the advice received to the action taken. In addition, most users had accessed advice before becoming ill. Finally, users who were advised to contact NHS Direct may have been recommended by NHS Direct to contact their GP; however, this was not captured in either self-report data or patients’ GP notes.

Initial RCT uptake by patients was 6.95% (3044/43,769), suggesting that participants were more willing to engage in this type of research and may not be representative of the wider population. Therefore, participant and nonparticipant characteristics were compared for the RCT analysis, and as the index for multiple deprivation showed that participants were less deprived than the wider population, the RCT results were controlled for this variable. However, as the process evaluation was a secondary analysis of the data collected in the RCT, identifiable details (ie, home address) were removed from usage data; as a consequence, it was not possible to control for possible effects that lower levels of deprivation may have had on behavioral engagement.

*Internet Dr* was developed and trialed >10 years ago, and since that time, digital intervention technology has advanced considerably. In addition to the recommendations from the usage analysis, before further dissemination activities are undertaken, the intervention would need further testing and development to ensure that current accessibility guidelines are met and the content is mobile friendly.

### Conclusions

The findings from the process evaluation demonstrate the advantages of using systematic methods for analyzing digital intervention usage. By identifying specific metrics that are meaningful to the intervention structure, theory-based content, and target behavior, it was possible to examine how the intervention was effective, for whom, and in what context, and to provide specific recommendations for improving intervention design and implementation. Inferential analyses of usage identified that viewing advice pages from the *Internet Dr* intervention is effective at increasing the enablement of self-care for the symptoms of RTIs. Having identified content that is crucial for behavior change, this provides the opportunity to ensure that prior compulsory pages are streamlined to maximize the number of users reaching these *active ingredients,* thereby minimizing attrition. However, streamlining within components does not necessarily mean reducing the number of components available as users used the choice. These findings suggest that viewing advice pages before having an RTI encourages users to self-care for future symptoms. This means that for *Internet Dr*’s dissemination, users may be encouraged to access the intervention at their convenience rather than wait for the occurrence of an illness. The intervention was effective at reaching the target population of users who had previously failed to self-care for their symptoms and consulted their GP. Taking these findings into consideration, *Internet Dr* provides a model for future digital interventions aiming to increase self-care for other minor ailments.

## Appendix

Multimedia Appendix 1 Analyzing and Measuring Usage and Engagement Data framework, stage 1 checklist: familiarization with the data. Completed for Internet Dr.

Multimedia Appendix 2 Analyzing and Measuring Usage and Engagement Data framework, stage 2 checklist: selecting use variables and generating research questions. Completed for Internet Dr.

Multimedia Appendix 3 Analyzing and Measuring Usage and Engagement Data framework, stage 3 checklist: preparation for analysis. Completed for Internet Dr.

Multimedia Appendix 4 Psychological measures collected on the web at baseline and follow-up.

## Abbreviations

AMUsED: Analyzing and Measuring Usage and Engagement Data
GP: general practitioner
HCP: health care professional
MD: mean difference
NHS: National Health Service
NIHR: National Institute for Health Research
PBC: perceived behavioral control
PEI: patient enablement index
PETS: Problematic Experiences of Therapy Scale
RCT: randomized controlled trial
RTI: respiratory tract infection
TPB: theory of planned behavior
