# Supplementary material for: A Digital Intervention for Respiratory Tract Infections (Internet Dr): Process Evaluation to Understand How to Support Self-care for Minor Ailments
Source: JMIR Form Res. 2022 Jan 19;6(1):e24239. doi: 10.2196/24239 (PMC8811700; doi:10.2196/24239)
Supplement: Multimedia Appendix 1 [file formative_v6i1e24239_app1.pdf]

AMUsED Framework: Assessing and Measuring Usage and Engagement Data – Stage 1 Checklist

Familiarisation with the data – identifying variables	Intervention Name: *Internet Dr*
Generic questions by data type	Intervention Details (including dataset)
**1. Intervention characteristics. Data for intervention architecture and content.**	
**1.1. Workflow. Intervention structure and expected participant interaction and navigation through the intervention.**	
How many logins/sessions are available?	*Not structured as sessions, access intended during an RTI.*
When are they available?	*All content available at every access. Prompted logins at: baseline, 5 x 4-weekly interim questionnaires, & follow-up questionnaires. Then during illness and 48 hour follow-up.*
Are new sessions released depending on time elapsed or task-completion?	*No.*
Are there limitations on the availability of the intervention?	*No.*
Is the purpose of the session to collect data and/or use the intervention?	*Questions are asked after login to ascertain user's purpose – either to use intervention for illness or to complete interim questionnaires.*
When is the intervention considered to be finished?	*The study finishes after the follow-up questionnaire at 24 weeks. The intervention was finished when users no longer wished to access it.*
What prompts are used to encourage usage (e.g. emails, texts, notifications) and when are they sent?	*Email prompts for 4 weekly data collection. Additional emails are sent after logging in during illness to prompt repeat login after 48 hours.*
Does the intervention contain 'tunnelled' (compulsory) sequences of pages which users have to view in order to move forward?	*'Doctor's Questions' component is tunnelled as symptoms are assessed before providing illness management advice.*
Are users able to select linked sections they wish to view, and avoid others?	*There are 3 linked menu components available from the home page.*
**1.2. Content. Content available within the pages of the intervention.**	
What are the linked menu sections available?	*'Doctor's Questions'. Split into cough, sore throat, runny nose & fever. Asks about symptoms & provides advice recommending either: self-management, phoning NHS Direct for more advice, or seeking immediate attention via NHS Direct.*<br>*'Treatment Options'. Split into cough, sore throat, runny nose & fever. Advice on coping with symptoms: without medication, with medication, and boosting immune system.*<br>*'Common Questions'. 2 sets of FAQs: 'Ask the Internet Dr' - medical*

	questions about illness and treatment, 'Common myths about Colds and Flu' - general questions about illness beliefs.
What is the aim of each section and are they based on underlying theoretical constructs?	*Doctor's Questions and Common Questions support users who are unsure if their symptoms are serious and whether they need medical treatment. Based on Leventhal's Common Sense Model of Self-regulation of health and illness [1] to increase understanding of illness.*<br>*Treatment Options supports management of distressing symptoms. Based on Bandura's Social Cognitive Theory [2] to increase self-efficacy.*
In what order is it anticipated the sections will be used?	*No order is necessary but it is anticipated that Doctor's Questions will be viewed first as it is listed first.*
What features are available (e.g. forums, videos, printable information)? How long should they take to complete?	*Videos available: Welcome video on home page: 1 minute, 23 seconds, glands: 48 seconds, meningitis/septicaemia: 1 minute, 1 second, video sinusitis: 30 seconds. Printable material available but log-data doesn't record whether it was used.*
Are all sections/features available to all users throughout the intervention or are some tailored for specific times or users?	*Doctor's Questions and Treatment Options are split into cough, sore throat, runny nose & fever.*
Which pages are for data collection or to support use of the intervention, such as questionnaires or administration of the intervention (e.g. login, password change)?	*See Figure 1 for intervention flow chart showing content by individual page.*
Are there specific pages to mark the start and end of sessions?	*Home page is the first page visited.*
Which pages contain BCTs, such as information, planning, feedback etc...?	*See components listed above.*
What are the underlying BCTs associated with the page?	*N/a*
In which sessions are they available?	*See components listed above.*
Can specific BCTs be identified on particular pages or groups of pages? How many groups are there?	*Information on symptoms is collected in Doctors Questions.*
Do any of the pages have response options to collect additional information? What data is collected?	*See components listed above.*
**2. Accrued data. Data collected during an intervention.**	
**2.1. Self-report. Users' self-reported responses collected across various stages of the trial.**	
When are self-report questionnaires collected (e.g. weekly logins, monthly symptom information, follow-up at 6 months)?	*Baseline, every 4 weeks up to 24 weeks, during illness and 48 hour follow up.*
What demographic information is available (e.g. age, gender,	*Baseline: Age, gender, qualification, household, smoker, alcohol, ethnicity.*

education)?	
Which measures are specifically related to the target behavior and how often are they collected?	*During illness, every 4 weeks & follow-up (see also external data): GP visits & illness occurrence.* *Follow-up: Patient enablement index* [3]
Which measures of beliefs influential on the target behavior are collected and when?	*Baseline & follow-up: health locus of control* [4]*, Krantz health opinion survey* [5]*, TPB* [6]*. When ill & 48hr: IPQ-R* [7]*, TPB* [6]*.*
Are measures collected for health (e.g. conditions which may impact on target behavior or are co-morbid) and psychosocial factors (e.g. anxiety, illness perception, motivation)?	*Baseline, when ill & 48 hour, & follow-up: (Physical) Mobility, self-care, usual activities, pain, anxiety/depression. Baseline (see also external data): health anxiety inventory* [8]*.*
Are additional measures collected at follow-up (e.g. satisfaction, adherence)?	*Follow-up: website satisfaction, problematic experiences of therapy scale* [9]*.*
**2.2. Log-data. Information automatically collected through engagement with an intervention.**	
What data is the software platform able to record?	*Time and date, pages viewed & order, time spent on pages, self-report measures.*
Are number, date and time of logins available by individual user?	*Yes.*
Are individuals' total durations of usage accessible?	*Need to be extracted.*
Are the number and time of usage prompts recorded?	*Overall scheduled timings for emails are available, but not sent times by individual.*
Are there details for which pages were viewed, the sequential order and time spent viewing?	*Yes.*
**2.3. External data. Data collected independently but alongside intervention usage.**	
How and where is the data collected?	*Hand collected by the research team from users' GP notes.*
What data is collected?	*Number of GP visits for RTI during trial, for year prior to trial, and co-morbid illnesses. Antibiotic prescriptions for RTI.*
Which of these measures relate to or may impact on the target behavior?	*Number of GP visits for RTI during trial, for year prior to trial, and co-morbid illnesses.*
**3. Contextual data. Data indirectly related to the running of the intervention which may be influential over usage and analysis.**	
**3.1. External factors. Structures and events which may influence participation in the intervention.**	
How are users recruited to the intervention?	*Recruited via GP.*
Did any specific large-scale events, with the potential to impact on the intervention, occur during the period of the intervention?	*n/a*
**3.2. Previous theory and findings. Results of behavioral analyses carried out during intervention development (e.g. logic models), and analyses of clinical outcomes if available.**	

What are the hypothesised mechanisms of the intervention (e.g. as specified in the intervention's logic model)?	*Viewing Doctor's Questions and Common Questions will raise users' understanding of their symptoms, leading to them being less likely to consult their GP.*<br>*Viewing Treatment Options will raise users' self-efficacy in their ability to self-manage their illness, leading to them being less likely to consult their GP.*<br>*Level of anxiety may be associated with usage.*
Which factors are identified as important in qualitative research, and can they be related to the variables collected in the trial (e.g. preferences for specific pages)?	*N/a*
Which variables are identified as relating to outcomes (e.g. behavioral determinants, theoretical constructs, health factors)?	*Analysis of the data from the RCT focused on the outcomes of GP contacts and antibiotic use* [10]*. No analyses were carried out for usage, behavioral determinants or personal characteristics.*

1. Leventhal HA, Brissette I, Leventhal EA. The common-sense model of self-regulation of health and illness. In: LD Cameron, HA Leventhal (Eds), *The self-regualtion of health and illness behavoiur*. London, UK: Routledge; 2003:42-65. ISBN: *9780415297011*
2. Bandura A. *Self-Efficacy: The Exercise of Control*. New York, US: WH Freeman; 1997. ISBN: 9780716728504
3. Howie JG, Heaney DJ, Maxwell M, Walker JJ. A comparison of a Patient Enablement Instrument (PEI) against two established satisfaction scales as an outcome measure of primary care consultations. *Fam Pract*. 1998;15(2):165-171. PMID: 9613486
4. Wallston KA, Wallston BS, DeVellis R. Development of the Multidimensional Health Locus of Control (MHLC) scales. *Health Educ Monogr*. 1978;6(2):160-170. doi:10.1177/109019817800600107
5. Krantz DS, Baum A, Wideman MV. Assessment of Preferences for Self-Treatment and Information in Health Care. *J Pers Soc Psychol*. 1980;39(5):977-990. PMID: 7441487
6. Ajzen I. The theory of planned behavior. *Organ Behav Hum Decis Process*. 1991;50(2):179-211. doi:10.1016/0749-5978(91)90020-T
7. Moss-Morris R, Weinman J, Petrie K, Horne R, Cameron L, Buick D. The Revised Illness Perception Questionnaire (IPQ-R). *Psychol. Health.* 2002;17**;**1–16. Doi:10.1080/08870440290001494

8. Salkovskis PM, Rimes KA, Warwick HMC, Clark DM. The Health Anxiety Inventory: development and validation of scales for the measurement of health anxiety and hypochondriasis. *Psychol Med*. 2002;32(5):843-853. PMID: 12171378

9. Kirby S, Donovan-Hall M, Yardley L. Measuring barriers to adherence: validation of the problematic experiences of therapy scale. *Disabil Rehabil*. 2014;36(22):1924-1929. PMID: 24410171

10. Little P, Stuart B, Andreou P, McDermott L, Joseph J, Mullee M, Moore M, Broomfield S, Thomas T, Yardley L. Primary care randomised controlled trial of a tailored interactive website for the self-management of respiratory infections (Internet Doctor). *BMJ Open*. 2016;6(4):e009769. PMID:27098821
