# Supplementary material for: A Digital Intervention for Respiratory Tract Infections (Internet Dr): Process Evaluation to Understand How to Support Self-care for Minor Ailments
Source: JMIR Form Res. 2022 Jan 19;6(1):e24239. doi: 10.2196/24239 (PMC8811700; doi:10.2196/24239)
Supplement: Multimedia Appendix 2 [file formative_v6i1e24239_app2.pdf]

AMUsED Framework: Analysing and Measuring Usage and Engagement Data – Stage 2 Checklist

Selecting usage variables and generating research questions	Intervention Name: Internet Dr
Generic questions	Intervention Specific
1. **Descriptions of usage variables. Which usage variables are relevant to the intervention and in which format (e.g. number of users/sessions, duration, percentage of total, dichotomous)?**	
Completing intervention/trial period (stage1; 1.1 & 2.2). E.g. How many users complete the trial? What is the average time period taken to complete?	*Number of users completing follow-up measures at 24 weeks.*<br>*Number of users ill during trial period (logged in during illness, self-report at 4 week measures, from GP notes).*<br>*Total number of pages viewed across the trial.Total time spent on pages*
Logins or sessions where the intervention was accessed (stage 1; 1.1 & 2.2). E.g. How many users start/complete each login/session? How long does it take to complete each session? How many pages are viewed within the session? Which session has the highest proportion of pages viewed, or duration of time spent on it?	*Purpose of login: to complete baseline, 4 weekly measures or manage illness.*<br>*Content viewed, number of pages and time spent when using intervention during illness.*
Date of login and usage. E.g. When do users login? What time of year? Are there changes in frequency of logins?	*n/a*
Time of day of login and usage. E.g. What time of day is usage? Are users more likely to spend longer on the intervention at certain times?	*Proportion of usage outside vs during GP hours.*
Days/weeks of usage (stage1; 1.1 & 2.2). E.g. For how many days/weeks out of the total is the intervention accessed for? How many times within a week is the intervention accessed? Are there repeated uses within the same day?	*Number of times intervention content was accessed.*
Response to prompts/notifications (e.g. requests to login, email, text, upload data) (stage1; 1.1 & 2.2). E.g. How many responses are sent? How long after receiving notification do users take to log-in or respond?	*Number of users who logged in to illness follow-up at 48hr.*
Features/menu sections used (stage 1; 1.1, 1.2 & 2.2). E.g. How many features/sections are accessed? How many users access each feature/section? Which sections/features are completed and by how many users? Which feature/section has the highest proportion of	*Number of users who viewed each of the components.*<br>*Component most frequently viewed first.*<br>*Component most frequently viewed first during a login for illness.*<br>*Doctors Questions: Dropout by page. Number of users moving back and*

pages viewed? Which feature/section has the highest proportion of time spent on it? What order are the features/sections viewed in? Is this the anticipated order? Which features/sections have the highest proportion of drop-out?	*forwards through pages. Split of users by symptom. Number of users who saw the advice pages, and type of advice given.* *Treatment Options: Number of pages viewed. Time spent in this section. Number of users who viewed each of the treatment options. Split of users by symptom.* *Common Questions: Number of pages viewed. Time spent in this section. Number of users who viewed the two sections, and each of the questions.* *Number of users viewing each video. Time spend on the video.*
Revisiting sections/features (stage 1; 1.1, 1.2 & 2.2). E.g. Are sections used repeatedly? How many times are they revisited, and for how long? Which sections/features are most revisited?	*Number of users re-visiting each component and frequency of revisits.* *Number of re-visits during illness.* *Number of users revisiting sections with the same symptoms/different symptoms.* *Number of users who login when ill more than once.*
Type of content/BCTs used (excluding administration pages) (stage 1; 1.2 & 2.2). E.g. How many groups of pages with similar content are accessed and by how many users? How many pages within the group are used? How many users view each page? Which groups of pages have the highest proportion of views? Which pages are viewed at each login, and when is the largest amount of pages viewed? Which pages have higher drop-out?	*See features/menu sections*
Completing ongoing measures (e.g. monthly questionnaires, response options within content pages, uploading information or text responses) (stage 1; 2.1 & 2.2). E.g. How many users complete ongoing measures? When do they complete them? Do they also access the intervention during that session?	*Number of users logging in when ill, and also viewing content at that time.*
External device usage (e.g. wearables and other sensor technologies) (stage 1; 2.3). E.g. How much time is spent with the device? How many times is it used? What number of days/weeks is it used for?	*n/a*
**2. Relationships between usage and participant characteristics. Are user's demographic, physical or psychosocial characteristics at baseline related to intervention usage?**	
Are any characteristics at baseline related to usage? E.g. Is education level associated with number of pages viewed? Is anxiety associated with revisiting features? Is current health related to usage of external	*Are age or gender associated with different patterns of usage?* *Is level of anxiety at baseline associated with different patterns of usage?*

devices? Are users who spend more time on the intervention older than those who spend less time? Which characteristics are associated with drop-out?	*Is having visited a GP in the past year associated with usage?*
Are any contextual factors related to usage (stage1; 3)? E.g. Does manner of recruitment relate to usage?	n/a
Do high/low users differ by other usage factors? E.g. Do users with higher levels of usage view more types of content than users with lower levels? Do users who complete more sessions also access a specific feature more often? Is usage of an external device related to intervention usage?	*Is logging in when ill related to content previously viewed?*<br>*Do users who self-report GP visits use the intervention differently to users who visited their GP but didn't report it?*
**3. Relationships between usage, target behaviours and behavioural determinants. Which usage variables are associated with follow-up measures for target behaviour and behavioural determinants? Which usage variables help explain changes in behaviour across the intervention?**	
Are baseline measures for target behaviour and behavioural influencers related to usage? E.g. Do users with low target behaviour spend less time on the intervention? Do users with high target behaviour revisit a specific feature? Is the number of days the intervention is used for related to a behavioural determinant?	*Are measures of believed necessity for HCP at baseline associated with usage?*<br>*Are measures of believed necessity for HCP associated with usage during the period of illness (e.g. viewing content, completing 48hr follow-up).*
Which usage variables are related to target behaviours and behavioural determinants at follow-up? E.g. Is completing/not completing a particular section associated with target behaviour at follow-up? Is the time spent on a session related to target behaviour? Do users with high target behaviour at follow-up complete a specific section compared to users with low target behaviour? Do users who view a group of pages containing a specific BCT score higher/lower for the associated behavioural determinant?	*Is usage related to enablement? Is logging in when ill related to enablement scores?*<br>*Is usage associated with the amount of times a user is ill during the trial?*<br>*Are changes in measures of believed necessity for HCP across the study associated with usage?*
Is usage associated with measures for satisfaction at follow-up? E.g. Are high levels of satisfaction associated with accessing more pages? Do users with low satisfaction spend less time using external devices?	*Are scores for website satisfaction or usage experience associated with usage?*
Do users who report positive changes in target behaviour/behavioural determinants from baseline to follow-up use the intervention differently to those who do not? E.g. Do users who report positive behaviour change spend more time on the intervention? Do users	*Did users with lowered beliefs in necessity of HCP use the intervention differently?*<br>*Did users who reported increased positive beliefs at 48hr illness follow-up view different content during illness?*

who report increases in a behavioural determinant view more pages from a specific section containing a relevant BCT?	
Are relationships between usage and outcome measures moderated by demographic, psychosocial or health factors? E.g. Is the relationship between time spent on the intervention and behaviour altered when moderated by anxiety?	*Does anxiety moderate relationships between usage and enablement?* *Does age moderate the relationship between usage and enablement?*
What level of usage is necessary for 'effective engagement'? E.g. Do outcome measures plateau after viewing certain content, or after a certain amount of time or sessions completed?	*Did users need to see a specific amount or section of content, at a certain time in order to change their behaviour?*
