# Supplementary material for: A Digital Intervention for Respiratory Tract Infections (Internet Dr): Process Evaluation to Understand How to Support Self-care for Minor Ailments
Source: JMIR Form Res. 2022 Jan 19;6(1):e24239. doi: 10.2196/24239 (PMC8811700; doi:10.2196/24239)
Supplement: Multimedia Appendix 3 [file formative_v6i1e24239_app3.pdf]

AMUsED Framework: Assessing and Measuring Usage and Engagement Data – Stage 3 Checklist

Preparation for analysis	Intervention Name:
Generic questions	Intervention Details
**1. Resources**	
What is the timeframe for completing the analyses?	*August 2019*
What resources are needed? E.g. additional research time, expertise.	*Expertise on types of analyses, confounding variables (e.g. co morbid illnesses), and moderators. Support with visualisation tool, and help extracting data.*
Is a plan of analysis already available? How does the analysis plan developed using the framework compare to that plan? Are changes or updates needed?	*Primary analyses have already been carried out (see stage 1, 3.2), no further plan is available.*
**2. Selecting types of analysis and analytical software**	
Will the usage data be triangulated with qualitative data?	*No*
Is there sufficient statistical power to answer the planned research questions?	*Analyses using whole intervention group are sufficiently powered, sub-group analyses may not be.*
What analytical tools are available?	*SPSS and LifeGuide Visualisation Tool (LVT)*
Can the selected measures of usage be analysed using the available tools? Is bespoke software necessary (e.g. visualisation techniques)?	*Sequence of use of sections and movement through Doctors Questions will need to be analysed using LVT*
**3. Data preparation**	
When is the data available?	*Now*
How many datasheets are there? Will these need to be amalgamated?	*7 datasheets: Log data (session details, page flow, page durations, user data), GP notes, two SPSS datasheets from previous analysis.*<br>*LVT: Need individual sheets for LVT. Session details must contain important self-report measures from user data. Most measures have already been saved, any measures outstanding will need to be copied across. Need primary outcome measures, previous behaviour and co morbid illnesses from GP notes.*<br>*SPSS: all data for analysis must be available on one data sheet. The two previous SPSS sheets contain some but not all measures relevant to usage. Relevant measures from GP notes will be copied to session details, along with usage variables from user data and page*

	durations.
What format is the datasheet in (e.g. excel, .csv)? Will it need converting for analysis?	*Log data (session details, page flow, page durations, user data) is excel. These will need to be converted from worksheets to .csv for LVT.*<br>*GP notes SPSS.*<br>*SPSS datasheets from previous analysis.*
Is the data structured to work with the tools available? What changes or data cleaning are needed?	*Data has already been collected and is compatible with LVT.*
What preparation does the data need (e.g. cleaning, anonymising)?	*Data needs cleaning and anonymising.*
Are all variables readily available or will they need extracting/transforming/recoding?	*In the user data datasheet each login is recorded on a new row so that a user who has logged in 7 times will have 7 rows of data. However, in the session details datasheet each user has only 1 row of data. To make it possible to copy variables across from user data to session details, they must first be transformed into 1 row of data. Code will be needed to extract data for total intervention time and pages used, and temporal analyses.*
Is the data in the right format to answer the research questions? Will it need adapting (e.g. continuous variables changed to categorical)?	*Behavioural constructs are measured as continuous variables. For comparisons of usage by high/low groups these will need to be changed to categorical.*
