# Supplementary material for: A Digital Intervention for Respiratory Tract Infections (Internet Dr): Process Evaluation to Understand How to Support Self-care for Minor Ailments
Source: JMIR Form Res. 2022 Jan 19;6(1):e24239. doi: 10.2196/24239 (PMC8811700; doi:10.2196/24239)
Supplement: Multimedia Appendix 4 [file formative_v6i1e24239_app4.pdf]

Multimedia Appendix 4: Psychological measures collected online at baseline and follow-up.

| Collected online across trial period | | | |
|---|---|---|---|
| ***Measure*** | ***Description*** | ***Baseline*** | ***24 weeks*** |
| Health Anxiety Inventory[21] | 14 items, 4 response options numbered 0-3, where 3 is highest level of anxiety (eg, I do not worry about my health/I spend most of my time worrying about my health). | ✓ | |
| Health Locus of Control, powerful others B scale[22] | 3 items, 0-7 Likert scale ranging from ‘strongly agree’ to ‘strongly disagree’ where low scores indicate high external locus of control/greater reliance on HCP (eg, If I see an excellent doctor regularly, I am less likely to have health problems). | ✓ | ✓ |
| Krantz Health Opinion Survey, behavioral involvement scale[23] | 7 items. 0-7 Likert scale ranging from ‘strongly agree’ to ’strongly disagree’. Reverse scored where necessary so that low scores indicate stronger reliance upon HCP (eg, Learning how to cure some of your own illness without contacting a physician may create more harm than good). | ✓ | ✓ |
| Theory of Planned Behaviour (TPB)[24] | *Attitudes*. 2 items. 2 response options (eg, Getting and following advice from this website will be/was: useful/useless). | ✓ | ✓ |
| | *Subjective Norms*. 2 items, 0-7 Likert scale, ranging from ’strongly disagree’ to ’strongly agree’, where high scores indicate positive norms to use and follow the website advice (eg, My family and friends think that it is/was sensible for me to get and follow advice from this website). | ✓ | ✓ |
| | *Perceived Behavioral Control (PBC)*. 2 items, 0-7 Likert scale, ranging from ’strongly disagree’ to ’strongly agree’, where high scores indicate positive beliefs to use and follow the website advice (eg, It will be/was easy for me to get/follow advice from this website). | ✓ | ✓ |
| | *Intentions*. 2 items, 0-7 Likert scale, ranging from ’strongly disagree’ to ’strongly agree’, where high scores indicate higher intentions to use and follow the advice (eg. I intend to get and follow advice from this website). | ✓ | |
| Problematic Experiences of Therapy Scale (PETS)[25] | *Symptoms*. 3 items, 1-5 Likert scale ranging from ‘agree strongly’ to ‘disagree strongly’, where low scores indicate a greater degree of problem (eg, I did not use/follow the Internet Doctor advice because it made my symptoms worse). | | ✓ |
| | *Uncertainty about how to use the intervention*. 3 items, 1-5 Likert scale ranging from ‘agree strongly’ to ‘disagree strongly’, where low scores indicate a greater degree of problem (eg, I could not use/follow the Internet Doctor advice because I was unsure how to do it properly). | | ✓ |
| | *Doubts about intervention efficacy*. 3 items, 1-5 Likert scale ranging from ‘agree strongly’ to ‘disagree strongly’, where low scores indicate a greater degree of problem (eg, I did not use/follow the Internet Doctor advice because I was not sure if it was helping). | | ✓ |
| | *Practical problems*. 5 items, 1-5 Likert scale ranging from ‘agree strongly’ to ‘disagree strongly’, where low scores indicate a greater degree of problem (eg, I found it difficult to remember to use/follow the Internet Doctor advice). | | ✓ |
| Patient Enablement Index | 6 items. Thinking about the kinds of symptoms we have asked about in this study (eg, cough, sore throat, sinus pain, runny nose, cold and flu | | ✓ |

| | |
|---|---|
| (PEI)[8] | symptoms), compared with before you took part in this study, do you feel you are:<br>Able to cope with life when you have these symptoms?<br>Able to understand these symptoms?<br>Able to cope with these symptoms?<br>Able to keep yourself healthy?<br>Confident about your health when you have these symptoms?<br>Able to help yourself when you have these symptoms?<br>0-2 Likert scale, where 0 = 'same or less', 1 = 'better', 2 = 'much better'. High scores indicate better enablement. |
